# Influence of Growth Rate and Magnetic Field on Microstructure and Properties of Directionally Solidified Ag-Cu Eutectic Alloy

**DOI:** 10.3390/ma9070569

**Published:** 2016-07-13

**Authors:** Xiaowei Zuo, Congcong Zhao, Lin Zhang, Engang Wang

**Affiliations:** 1 Key Laboratory of Electromagnetic Processing of Materials (Ministry of Education), Northeastern University, Shenyang 110004, China; congcongzhao2015@outlook.com (C.Z.); zhanglin@epm.neu.edu.cn (L.Z.); 2 School of Metallurgy, Northeastern University, Shenyang 110004, China; 3 School of Materials Science and Engineering, Northeastern University, Shenyang 110004, China

**Keywords:** Ag-Cu eutectic alloy, magnetic field, growth rate, eutectic lamellar spacing, microhardness, strength

## Abstract

We report the influence of growth rate and external magnetic field on the eutectic lamellar spacing and properties of directionally-solidified Ag-Cu eutectic alloys. The results indicated that the relationship between the lamellar spacing of directionally-solidified Ag-Cu alloys and the growth rate matched the prediction of the Jackson-Hunt model, and the constant was 5.8 µm^3^/s. The increasing external magnetic field during solidification tilted the growth direction of the lamellar eutectics, and coarsened the eutectic lamellar spacing. These decreased the microhardness and strength of Ag-Cu alloys, but increased their electrical conductivity. The competitive strengthening contributions between the refinement of the eutectic lamellar spacing and the change in growth direction of the eutectics resulted in higher strength in the as-rolled sample with a 0.8 T magnetic field than with other samples, which was confirmed from higher relieved deformation energy using differential scanning calorimetry.

## 1. Introduction

Eutectic alloys are important in casting, soldering and welding processes due to their low melting temperatures, good forming flexibility, and excellent mechanical properties [1]. These eutectic alloys, with lamellar, fibrous, or rod structures, can be produced by directional solidification. These structures are strongly dependent on the solidification parameters, which are determined by the material properties. The relationship between the eutectic microstructure and solidification conditions has been studied extensively, and a general quantitative equation was first proposed by Jackson and Hunt (J-H model) [2,3], which was based on a solution of the diffusion problem at the solid/liquid interface. They predicted that the product of the growth rate *V* and the square of the eutectic lamellar spacing (ELS) λ should be constant, i.e., λ^2^*V* = const., with the hypothesis that the interfacial undercooling was at a minimum as the eutectics were produced [2]. They concluded that the ELS is one of the most crucial microstructural parameters for eutectic alloys, and the growth rate is capable producing variations in both the liquid diffusion coefficient and the convection, changing the ELS correspondingly.

The external magnetic field (MF) has a similar effect on the eutectic solidification. Extensive experimental studies have been carried out to quantify the changes to the eutectic microstructure after subjecting alloys to MF during solidification. Li et al. [4] found that, at a low growth rate, MF decreased the eutectic spacing of the directionally solidified (DS) Al-Al_2_Cu eutectic alloy, and they posited that the change was a result of the decrease of the liquid diffusion coefficient induced by the external MF. Liu et al. [5] found that an applied MF during solidification of Al-Si alloys decreased the lamellar spacing of the eutectics by decreasing the diffusion at the solid/liquid interface. Li et al. [6,7] found that the ELS in Cu-Ag alloys was decreased after applying a 12-T MF. Li et al. [8], however, found that the application of a static MF to semi-continuous casting Al-Fe alloys increased the ELS due to the initiation of the secondary arms. Therefore, the effect of an external MF on the ELS is not entirely clear, and the properties of materials solidified with MF are rarely studied. 

In this paper, Ag-Cu, a representative eutectic system, was chosen for study, partly due to its wide applications in both DC and pulsed high-field magnets [9,10,11,12,13]. In previous publications [7,14,15,16,17], we introduced a MF during conventional solidification to fabricate high-performance Cu-Ag alloys, and the results showed that the ELS of hypoeutectic Cu-Ag alloys decreased due to the external MF application during solidification. The purpose of the present work is to investigate how the solidification growth rate and a MF applied in directional solidification influences the ELS of Ag-Cu alloys. Meanwhile, DS Ag-Cu alloys were prepared via cold rolling for comparison, to illustrate the differences between the resulting properties of Ag-Cu alloys.

## 2. Materials and Methods

**Figure 1:**
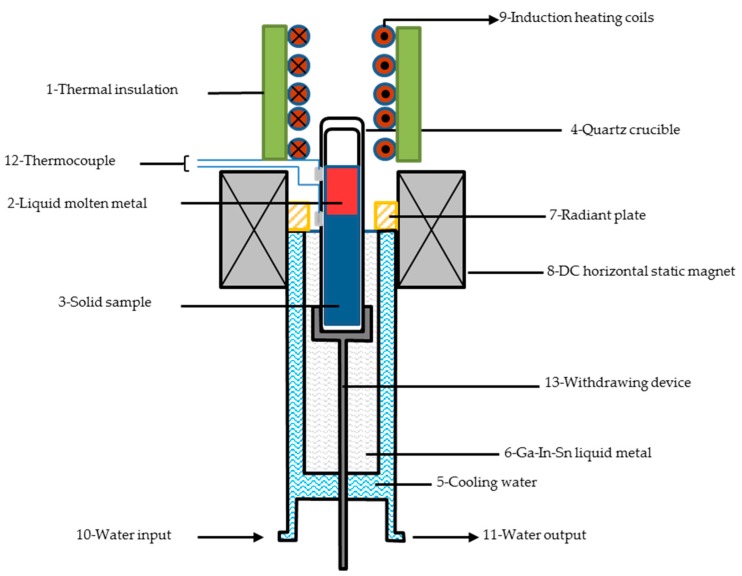
Schematic diagram of the directional solidification apparatus with a horizontal static MF.

Ag-28%Cu (weight percent, abbreviated as Ag-Cu in the following texts) eutectic master alloys were prepared by vacuum induction melting under argon atmosphere using oxygen-free Cu and electrolytic Ag rods. The polished sample, with a diameter of 9.5 mm and a height of 120 mm, was placed in a quartz crucible with a diameter of 10 mm. Together they were loaded in a directional solidification apparatus. The schematic diagram of the directional solidification equipment is illustrated in Figure 1, where a horizontal static MF up to 1.12 Tesla (T) was subjected to the liquid-solid interface of the molten metal. Solidification of the Ag-Cu eutectic alloy was carried out at a constant temperature gradient (4 K/mm) with different growth rates (50, 100, and 200 µm/s) under different horizontal MFs (0, 0.8, and 1.12 T). The detailed experimental protocol can be found in previous works [14,15]. The specimens with a thickness of 5 mm machined from the as-DS Ag-Cu alloys were then cold rolled to a total reduction of 94% without any heat treatment. The reduction is defined as 100 × (*t*_0_ − *t*)/*t*_0_%, where *t*_0_ is the initial thickness and *t* is the thickness after cold rolling. Differential scanning calorimetry (DSC, Shimadzu Corporation, Kyoto, Japan) analysis was carried out on the rolled samples under an argon atmosphere with a Shimadzu DSC-60. The calibration procedures for the DSC tests have been reported elsewhere previously [18].

The as-DS and as-rolled specimens were sectioned, polished, and etched in a solution of 120 mL H_2_O, 20 mL HCl, and 5 g FeCl_3_. The microstructure was observed by scanning electron microscope. The ELS of Ag-Cu eutectic alloys, λ, was measured on the longitudinal sections in 4–6 regions of the samples and analyzed quantitatively by the linear intercept method [19]. Microhardness measurements were performed with a standardized Vickers device using a 100 g load with a dwelling time of 10 s on the polished surfaces. The electrical resistivity of the samples was measured by the standard DC four-probe technique. The electrical conductivity was determined by using the standard conversion equation (IACS = the International Annealed Copper Standard, and 100% IACS = 1.7241 μΩ·cm). The tensile samples were machined to standard rectangular shape (width: 2 mm, gage length: 4 mm, thickness: less than 0.1 mm) according to the guidelines in ASTM E8-04, and tensile tests were performed on a MTS machine at a rate of 10^−3^·s^−1^ at room temperature.

## 3. Results and Discussion

**Figure 2:**
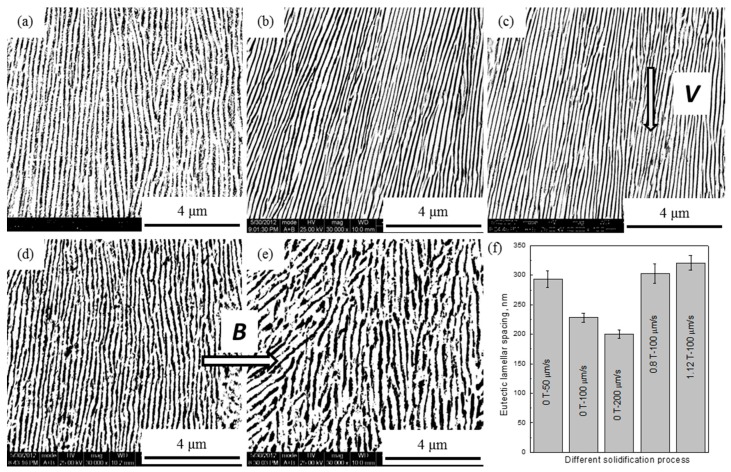
Microstructure of as-DS Ag-Cu eutectic alloys with different MFs (*B*) and different growth rates (*V*) at the constant temperature gradient of 4 K/mm. (**a**) 0 T and 50 µm/s; (**b**) 0 T and 100 µm/s; (**c**) 0 T and 200 µm/s; (**d**) 0.8 T and 100 µm/s; (**e**) 1.12 T and 100 µm/s; (**f**) the quantitative results of the ELS.

The effects of both the growth rate and the external MF on microstructural evolution of as-DS Ag-Cu eutectic alloys are shown in Figure 2. It indicates that the as-DS eutectic Ag-Cu alloys primarily consist of lamellar eutectic colonies, where dark lamellar structures are Cu phases, and light ones are Ag matrix phases [20,21]. With increasing growth rate (Figure 2a–c), the lamellar Cu gradually aligns along the growth direction and the eutectic spacing between Cu and Ag is refined. Some researchers [22] found that the crystallographic growth direction of Ag was <110>. Thus, we assumed that the increasing growth rate aligned Cu lamellar along the <110> crystallographic direction. The ELS of as-DS Ag-Cu eutectic alloys were statistically analyzed and the results are summarized in Figure 2f. The results show that the ELS of as-DS Ag-Cu eutectic alloys are about 200–320 nm (Figure 2a–c). With increasing growth rate in the absence of a MF, the ELS of Ag-Cu alloys was gradually decreased by 32% from 293 to 200 nm. This confirmed our previous thoughts that an increased growth rate would refine the ELS.

**Figure 3:**
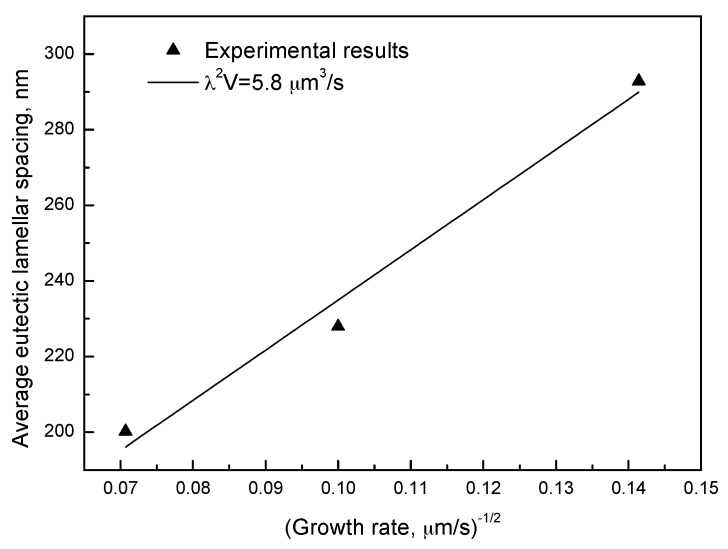
The ELS of as-DS Ag-Cu alloys as a function of the inverse square root of growth rate.

The effect of the growth rate, *V*, on the average ELS, λ, was previously studied in numerous eutectic lamellar systems and the experimental results generally follow the J-H model λ^2^*V* = constant [2]. The relationship between the ELS of Ag-Cu eutectic alloy and the growth rate agreed well with the J-H model, and the constant was found to be 5.8 µm^3^/s (Figure 3). We hypothesized that as the growth rate increased, the enriched solute in front of the lamellae increased. This resulting depression in front of Ag or Cu lamellae promoted the nucleation and subsequent growth of another Cu or Ag lamellae, which consequently decreased the ELS. The constant was different from that of 14 µm^3^/s, which was reported in previous works of Ag-Cu eutectic alloys by Cline et al. [22]. This deviation may have resulted from the refining effects from the melt. Intermedia induction heating (label 9 in Figure 1) instead of resistive heating was used to melt the DS samples, which induced electromagnetic forces during the melt. This force promoted the melt convection, which is called the electromagnetic stirring [23]. This might be responsible for the finer ELS in Ag-Cu eutectic alloy [22].

Even with an increase of the imposed MF (Figure 2b,d,e), for all tested growth conditions, the eutectic microstructure remained lamellar. However, the lamellar eutectic structure gradually degenerated, previously demonstrated in other work [4,24]. The external MF also made the lamellar Cu phases coarsen and distribute discontinuously. However, as the MF was increased to 0.8 T (Figure 2f) the ELS is increased by 33%, from 228 nm to 303 nm, but the ELS only changes slightly as the MF is increased further to 1.12 T. MF changed the growth direction of lamellar Cu (Figure 2d,e). In our previous work, we found that utilizing MF in solidification of DS Cu-6 wt %Ag alloy tilted the growth direction of Cu dendrite against the heat flow [15], and the angle between growth direction and heat flow direction agreed well with the equation proposed by Pocheau et al. [25]. We believe that the MF had an analogous impact on Cu lamellae and changed the growth direction of the Cu lamellar phase. We conclude that the imposed MF coarsened the eutectic lamellar structure and changed the growth direction of lamellar eutectics.

While the external MF and the growth rate during directional solidification affected the microstructures of Ag-Cu alloys, this influence was inversely related to the effect of the growth rates on the ELS and the lamellar structures. We hypothesized that the changes might be related to the effect of the MF on the liquid diffusion coefficient. The growth of the eutectic structure strongly depended on the solute diffusion in front of the liquid-solid interface [1]. The inter-lamellar spacing increased proportionally to the decreasing square root of the growth rate. Therefore, the changes of the eutectic inter-lamellar spacing were thought to be a result of the indirect effect of the MF on the growth rate in terms of diffusion. On the other hand, the melt convection affected the diffusion, resulting in the change to the ELS. There were two main mechanisms, which were recognized as effects between the MF and the electrical conductive melt. One was the electromagnetic damping effect, where an induced Lorentz force between MF and melt always tended to weaken convection, thereby damping the natural convection. When applying a MF to the directional solidification of eutectics, the decreasing convection slows down the solute diffusion at the solid-liquid interface, developing solute enriched layers, which in turn result in the decrease of the ELS. Another mechanism is the so-called thermoelectromagnetic convection (TEMC) effect [26,27,28]. During solidification, a thermoelectric current arises at the liquid-solid interface owing to the Seebeck effect. In the presence of an external MF, the direction of the electric current is changed and it is not uniformly parallel to the direction of the MF, which causes Lorentz forces within the solid-liquid zone, which might drive some motion. The influence of the high external MF on the melt convection was formulated by the Hartmann number. Applying the scale of the interlamellar spacings (about 0.2 µm) and the relative physical parameters [26], the Hartmann number is about 0.001 when imposed by an order of magnitude of 1 T MF. This indicated that the thermosolutal convection in molten metal, which was damped by the 1 T MF was negligible. However, at the scale of the interlamellar spacing, the TEMC was dominant and promoted the melt convection, which was consistent with previous reports [29,30]. Such weak convective flow gave rise to an increase of the ELS since the convection produced a slight shift of the solute concentration profile at the interface and a decrease of the eutectic growth undercooling [31]. Increasing convection was capable of increasing the atom diffusion in front of the liquid–solid interface, and coarsening the ELS of the Ag-Cu eutectic alloy. Therefore, we concluded that at the scale of the interlamellar spacing, TEMC rather than the electromagnetic damping effect was dominant, thus increasing the ELS of the Ag-Cu eutectic. Moreover, the increasing convection disturbed the preferential growth directions of the lamellar Cu phases, thus making lamellar Cu deviate from the preferred growth direction, which tended to align the orientation of Cu phases along the pulling direction.

**Figure 4:**
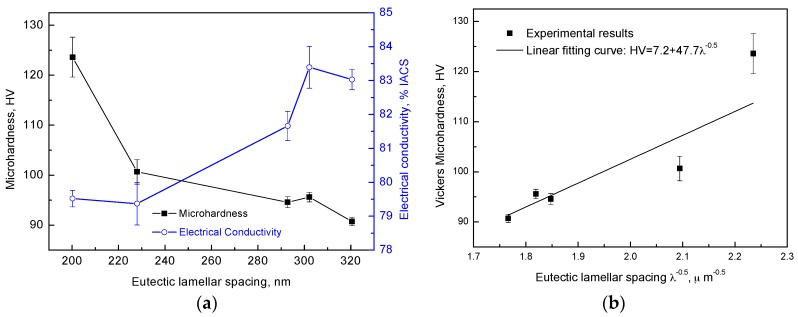
(**a**) Microhardness and electrical conductivity plotted as the ELS of DS Ag-Cu eutectic alloys with different MFs and growth rates; (**b**) Variation of microhardness as a function of the reciprocal square root of average interlamellar spacing for as-DS Ag-Cu alloy.

The microhardness and the electrical conductivity were measured and plotted as a function of the ELS in Figure 4a. The microhardness and the ultimate tensile strength (UTS), shown in Table 1, were decreased with increasing ELS. This was attributed to the Hall-Petch relationship between the microhardness and the characteristic microstructure, indicating that the increasing characteristic spacing decreased the strength [32]. The variations of the microhardness with the interlamellar spacing at a constant temperature gradient (*G* = 4 K/mm) are shown in Figure 4b. The value of hardness increased by increasing the value of λ^−0.5^. The relationship between hardness and the exponent function was obtained by using linear regression analysis, and the result was given as:

Hardness = 7.2 + 47.7 λ^−0.5^ (1)

Microstructure analysis (Figure 2) shows that the ELS decreases with decreasing growth rate, and, by increasing MF. Microhardness is increased with decreasing growth rate and by increasing MF according to Hall-Petch equation [32]. However, Figure 4a shows that the electrical conductivity of Ag-Cu alloys increases gradually with increasing ELS. Generally, the total resistivity of the metallic material is described by the sum of the individual contributions from phonons, impurities, dislocations, and interfaces [33]. Contributions from phonons, impurities, and scattering are assumed to be negligible if there are no other factors except the spacing. The effect of the size contribution to the resistance is defined as [31]:

(2) $$\rho =\rho_{0}\left[1+0.75\left(1-p\right)l_{0}/\lambda \right]$$

where the *l*_0_ is the mean free path of the electron in Ag at room temperature and *p* is the specularity factor which is in the range of 0.8–0.95 [12,34]. The external MF was incapable of changing *ρ*_0_, *p* or *l*_0_ because these were intrinsic physical factors of the elements [12,34]. As the ELS *λ* increased, electrical resistivity decreased and electrical conductivity increased, which was consistent with our results in Figure 4a.

Relative mechanical and electrical parameters of Ag-Cu alloys are presented in Table 1. It indicates that the external MF decreases the UTS of as-cast Ag-Cu alloys. With respect to the as-rolled samples, however, the sample with 0.8 T displays an increased UTS by 8% compared to that without MF.

**Figure 5:**
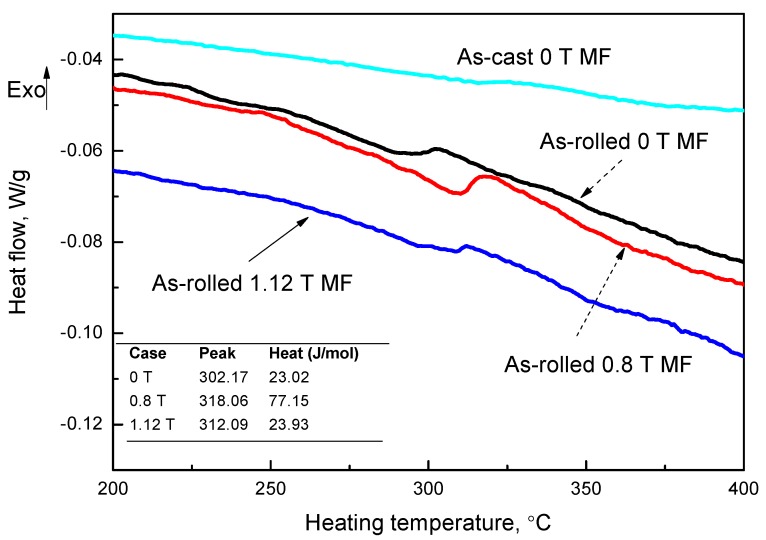
DSC curves of as-rolled Ag-Cu alloys at a heating rate of 10 K/min.

DSC results displayed in Figure 5 reveal that there are exothermal reactions during the heating process, which indicate the release of deformation energy because there are no peaks found in the as-cast sample. The sample with 0.8 T MF released more latent heat during the heating profile. Higher UTS was believed to be a result of the higher deformation energy, which confirmed our mechanical results (Table 1). Generally, electrical conductivity and UTS are trade-offs, which is consistent with the fact that the sample with 0.8 T MF in Table 1 has the lowest electrical conductivity. The lower conductivity might be a result of more interface scattering resistivity, which is found in the sample with higher strength.

The external MF changed the <110> growth direction and coarsened the ELS. During deformation, Cu and Ag have <110> textures [13]. The change in the growth direction might increase the barriers to form this kind of texture, thus increasing the strength. On the other hand, the coarsening ELS definitely decreased the strengthening according to Hall-Petch equation. The competitive strengthening between the ELS and the change in growth direction controlled the overall strength of the as-rolled Ag-Cu alloy. The change in the growth direction might be dominant, which was thought to be one of the reasons that the as-rolled sample with 0.8 T MF had a higher strength than the 0 T sample.

## 4. Conclusions

The influences of growth rate and external MFs up to 1.12 T on the ELS, mechanical, and electrical properties of DS Ag-Cu alloys were investigated.

(1) The relationship between the measured lamellar spacing of as-DS Ag-Cu eutectic alloys and growth rate followed the J-H model and the constant was found to be 5.8 µm^3^/s.
(2) The external MF changed the growth direction of the Ag-Cu lamellar eutectic and coarsened the spacing. It decreased the microhardness and UTS, but increased the electrical conductivity.
(3) The UTS of the as-rolled Ag-Cu sample with 0.8 T increased by 8% than that without MF. DSC results showed that higher deformation energy was released in the as-rolled sample with 0.8 T MF.
(4) The competitive strengthening between the coarsening ELS and the change in growth direction affected the strength. In our case, the change in growth direction might play a more important role in the strength of DS Ag-Cu alloys.

## Figures and Tables

**Table 1:** The mechanical and electrical properties of as-DS and as-rolled Ag-Cu alloys.

Specimen	Elongation	UTS	Yield Strength	Conductivity
	%	(M·Pa)	(M·Pa)	%IACS
0 T-As-DS	10.4%	349.1	310.9	79.4%
0.8 T-As-DS	14.0%	338.8	319.2	83.4%
1.12 T-As-DS	12.2%	263.9	261.0	83.0%
0 T-As-rolled	2.7%	854.8	818.3	62.88%
0.8 T-As-rolled	3.0%	949.5	884.8	53.36%
1.12 T-As-rolled	2.3%	874.5	840.4	56.68%

## References

[1] Kurz W., Fisher D.J. (1998). Fundamentals of Solidification.

[2] Jackson K.A., Hunt J.D. (1966). Lamellar and rod eutectic growth. Trans. Metall. Soc..

[3] Seetharaman V., Trivedi R. (1988). Eutectic growth: Selection of interlamellar spacings. MTA.

[4] Li X., Ren Z.M., Fautrelle Y. (2006). Effect of a high axial magnetic field on the microstructure in a directionally solidified Al-Al_2_Cu eutectic alloy. Acta Mater..

[5] Liu T., Wang Q., Zhang H.W., Lou C.S., Nakajima K., He J.C. (2011). Effects of high magnetic fields on solidification microstructure of Al-Si alloys. J. Mater. Sci..

[6] Li G.M., Liu Y., Su Y., Wang E.G., Han K. (2013). Influence of high magnetic field on as-cast structure of Cu-25 wt %Ag alloys. China Foundry.

[7] Li G.M., Wang E.G., Zhang L., Zuo X.W., He J.C. (2012). Effect of high magnetic field on solidified structure, drawn structure and electrical conductivity of Cu-25Ag alloys. Rare Metal Mater. Eng..

[8] Li L., Zhang Y.D., Esling C., Zhao Z.H., Zuo Y.B., Zhang H.T., Cui J.Z. (2009). Formation of twinned lamellas with the application of static magnetic fields during semi-continuous casting of Al-0.24 wt %Fe alloy. J. Cryst. Growth.

[9] Davy C.A., Ke H., Kalu P.N., Bole S.T. (2008). Examinations of Cu-Ag composite conductors in sheet forms. IEEE Trans. Appl. Supercond..

[10] Han K., Toplosky V., Goddard R., Lu J., Niu R.M., Chen J. (2011). Impacts of heat treatment on properties and microstructure of Cu16at%Ag conductors. IEEE Trans. Appl. Supercond..

[11] Zhao C.C., Zuo X.W., Wang E.G., Niu R.M., Han K. (2016). Simultaneously increasing strength and electrical conductivity in nanostructured Cu–Ag composite. Mater. Sci. Eng. A.

[12] Zuo X.W., Zhao C.C., Niu R.M., Wang E.G., Han K. (2015). Microstructural dependence of magnetoresistance in CuAg alloy solidified with high magnetic field. J. Mater. Process. Technol..

[13] Han K., Vasquez A.A., Xin Y., Kalu P.N. (2003). Microstructure and tensile properties of nanostructured Cu-25 wt %Ag. Acta Mater..

[14] Zuo X.W., Guo R., An B.L., Zhang L., Wang E.G. (2016). Microstructure, hardness and electrical resistivity of directionally solidified Cu-6%Ag alloy under a transverse magnetic field. Acta Metall. Sin..

[15] Zuo X.W., Guo R., Zhao C.C., Zhang L., Wang E.G., Han K. (2016). Microstructure and properties of Cu-6 wt %Ag composite thermomechanical-processed after directionally solidifying with magnetic field. J. Alloys Compd..

[16] Zuo X.W., Han K., Zhao C.C., Niu R.M., Wang E.G. (2014). Microstructure and properties of nanostructured Cu28 wt %Ag microcomposite deformed after solidifying under a high magnetic field. Mater. Sci. Eng. A.

[17] Zuo X.W., Zhao C.C., Wang E.G., Zhang L., Han K., He J.C. (2013). Microstructure evolution of the proeutectic Cu dendrites in diamagnetic Cu-Ag alloys by electromagnetic suppressing convection. J. Low Temp. Phys..

[18] Niu R.M., Han K., Su Y.F., Salters V.J. (2014). Atomic-scale studies on the effect of boundary coherency on stability in twinned Cu. Appl. Phys. Lett..

[19] Fan J.L., Li X.Z., Su Y.Q., Guo J.J., Fu H.Z. (2012). Effect of growth rate on microstructure parameters and microhardness in directionally solidified Ti-49Al alloy. Mater. Des..

[20] Tian Y.Z., Zhang Z.F. (2012). Bulk eutectic Cu-Ag alloys with abundant twin boundaries. Scr. Mater..

[21] Tian Y.Z., Li J.J., Zhang P., Wu S.D., Zhang Z.F., Kawasaki M., Langdon T.G. (2012). Microstructures, strengthening mechanisms and fracture behavior of Cu-Ag alloys processed by high-pressure torsion. Acta Mater..

[22] Cline H.E., Lee D. (1970). Strengthening of lamellar vs. Equiaxed Ag-Cu eutectic. Acta Metall..

[23] Griffiths W.D., McCartney D.G. (1996). The effect of electromagnetic stirring during solidification on the structure of Al-Si alloys. Mater. Sci. Eng. A.

[24] Li X., Fautrelle Y., Ren Z.M. (2008). Morphological instability of cell and dendrite during directional solidification under a high magnetic field. Acta Mater..

[25] Pocheau A., Deschamps J., Georgelin M. (2007). Dendrite growth directions and morphology in the directional solidification of anisotropic materials. JOM.

[26] Lehmann P., Moreau R., Camel D., Bolcato R. (1998). A simple analysis of the effect of convection on the structure of the mushy zone in the case of horizontal Bridgman solidification-comparison with experimental results. J. Cryst. Growth.

[27] Li X., Fautrelle Y., Ren Z.M. (2007). Influence of an axial high magnetic field on the liquid-solid transformation in Al-Cu hypoeutectic alloys and on the microstructure of the solid. Acta Mater..

[28] Li X., Fautrelle Y., Gagnoud A., Du D.F., Wang J., Ren Z., Nguyen-Thi H., Mangelinck-Noel N. (2014). Effect of a weak transverse magnetic field on solidification structure during directional solidification. Acta Mater..

[29] Zhang T., Ren W., Dong J., Li X., Ren Z., Cao G., Zhong Y., Deng K., Lei Z., Guo J. (2009). Effect of high magnetic field on the primary dendrite arm spacing and segregation of directionally solidified superalloy DZ417G. J. Alloys Compd..

[30] Li X., Ren Z., Wang J., Han Y., Sun B. (2011). Influence of a weak static magnetic field on the primary dendrite arm spacing of a directionally solidified Ni-based superalloy. Mater. Lett..

[31] Ma D., Jie W.Q., Li Y., Ng S.C. (1998). Effect of weak convection on lamellar spacing of eutectics. Acta Mater..

[32] Freudenberger J., Lyubimova J., Gaganov A., Witte H., Hickman A.L., Jones H., Nganbe M. (2010). Non-destructive pulsed field CuAg-solenoids. Mater. Sci. Eng. A.

[33] Verhoeven J.D., Downing H.L., Chumbley L.S., Gibson E.D. (1989). The resistivity and microstructure of heavily drawn Cu-Nb alloys. J. Appl. Phys..

[34] Heringhaus F. (1998). Quantitative Analysis of the Influence of the Microstructure of Strength, Resistivity, and Magnetoresistance of Eutectic Ag-Cu.

